# Ultrasound rejuvenation for upper facial skin: A randomized blinded prospective study

**DOI:** 10.1111/jocd.16482

**Published:** 2024-07-21

**Authors:** Wei Chen, Yuequ Deng, Guanqun Qiao, Wei Cai

**Affiliations:** ^1^ Department of Plastic Surgery The Second Affiliated Hospital of Nanjing Medical University Nanjing China

**Keywords:** brow lifting, focused ultrasound, tightening, upper facial rejuvenation

## Abstract

**Background:**

Growing demand for facial rejuvenation drives advancements in these therapies, including laser, radiofrequency, and focused ultrasound, alongside thermal stimulation adjuncts. These methods, known for stimulating collagen regeneration, skin tightening, and lifting, have gained popularity due to their minimal side effects, low trauma, and high safety, demonstrating favorable outcomes in clinical practice.

**Objective:**

We sought to assess the efficacy of ultrasound skin tightening for brow lift within the scope of a procedure addressing facial sagging across the entire face. Our aim was to explore a noninvasive method capable of effectively enhancing mild to moderate brow ptosis by tightening and lifting the skin in the upper facial region.

**Methods:**

This was a rater‐blinded, prospective cohort study. The upper facial region of the participants was treated with the new device, micro‐focused ultrasound (MFU), in model D3.0/D2.0/M3.0. Outcomes of brow lift were measured in comparison of pretreatment and posttreatment photographs and three‐dimensional (3D) vector analysis.

**Results:**

A total of 42 participants (37 females) were enrolled, with 2 participants withdrawing from the trial, resulting in 40 subjects who completed 180‐day‐follow‐up and evaluation. 35 (87.5%) were deemed to have clinically significant brow elevation by two blinded assessors (experienced clinicians) at 180‐day posttreatment (*p* < 0.01). The mean change in brow height after 90‐day was 2.16 ± 0.63 mm at the frontal position (straight‐ahead gaze) (*p* < 0.01). The 3D vector analysis reveals varying magnitudes of vector displacement in the upward and outward directions of the skin on the frontal region above the eyebrows.

**Conclusion:**

Focused ultrasound appears to be a safe and effective method for upper facial skin rejuvenation. A single focused ultrasound treatment on the forehead and temple areas resulted in an average brow elevation of 2.1 mm.

## 1 INTRODUCTION

Most cases of upper facial laxity are primarily attributed to aging, where the entire facial skin and superficial musculoaponeurotic system (SMAS) gradually undergo aging‐related relaxation and sagging with advancing age.^1^, ^2^ Other contributing factors include myasthenia gravis, facial paralysis, trauma, and congenital or hereditary conditions.^1^ Mild to moderate upper facial skin laxity can increase an appearance of facial aging and fatigue. The position of eyebrows plays a crucial role in upper facial aesthetics, while brow ptosis can be one of the earliest signs of facial aging.^3^ This issue receives significant attention in cosmetic surgery and many individuals seeking treatment often complain primarily of upper eyelid laxity. However, without adequate evaluation, performing upper eyelid skin excision surgery directly might exacerbate the degree of brow ptosis and further accentuate signs of facial aging.

Compared to invasive procedures or surgical interventions, noninvasive techniques show significant superiority in the treatment of skin tightening and lifting. They are characterized by minimal damage, rapid recovery, low risk, and fewer complications. Consequently, they are often referred to as “lunchtime procedures.”^4^ To meet the growing demand for these procedures, a variety of laser and radiofrequency devices have been developed, and applied to address issues such as skin wrinkles and tissue depression. Recently, micro‐focused ultrasound (MFU) has emerged as an effective noninvasive skin tightening technique. It can rapidly elevate tissue temperature to 60°C or even higher, targeting the dermal reticular layer and subcutaneous tissues to induce tightening effects, while minimizing damage to the dermal papillary layer and epidermis. The heat generated can penetrate through the dermis and subcutaneous tissue to reach the facial SMAS layer, causing immediate collagen denaturation and contraction, thereby reshaping and promoting the formation of new collagen.^5^, ^6^, ^7^

Although focused ultrasound has been approved and widely used for rejuvenating the mid and lower face as well as chin‐neck area, its effectiveness for upper facial and brow lifting is rarely reported. With the increasing demand for cosmetic procedures, focused ultrasound may be particularly suitable for addressing these specific indications. Recognizing the potential and applicability of noninvasive, nonablative skin tightening techniques, this study aims to maintain the tolerability and safety of the procedure while enhancing its effectiveness and durability. Hence, we introduce the use of focused ultrasound for treating comprehensive facial sagging while simultaneously addressing upper facial (forehead and temple areas) skin tightening.

## 2 METHODS

### 2.1 Patient selection

We conducted a randomized, rater‐blinded, prospective cohort study. It obtained approval from the Ethics Committee of the Second Affiliated Hospital of Nanjing Medical University. All participants were fully informed about the study and willingly agreed to comply with all requirements, volunteering to participate. All of them signed informed consent and were free to withdraw at any time during the clinical research (clinical trial ethic number: 2023‐XJ‐047‐01). The procedures adhered to the principles outlined in the Helsinki Declaration of 1964 and its subsequent revisions.

The study was performed at the Department of Medical Plastic and Cosmetic Medicine, the Second Affiliated Hospital of Nanjing Medical University from September 2023 to March 2024. We recruited a total of 42 informed participants, comprising 5 males and 37 females, with an average age of 41 years (ranged from 30 to 58 years). All participants exhibited mild upper eyelid skin laxity and brow ptosis. The evaluation was determined by two blinded plastic surgeons. Data were collected at four time points: before treatment, immediately after treatment, 90 days posttreatment, and 180 days posttreatment, based on the treatment schedule at enrollment.

#### 2.1.1 Inclusion criteria

Individuals aged 30–60 years with facial aging requiring treatment were included in this study.

#### 2.1.2 Exclusion criteria

The following were the exclusion criteria in this study: pregnant or lactating women; patients with severe organ system diseases, malignant tumors, or mental illness; individuals who underwent comprehensive facial plastic surgery or other cosmetic procedures (such as phototherapy, radiofrequency, botulinum toxin injections, hyaluronic acid fillers, etc.) in the 6 months prior to screening; acute or progressive skin diseases affecting the entire body or face (such as active acne, acute eczema, vitiligo, etc.); history of active facial infections, herpes, open wounds, streptococcal infections, or tumors; individuals with sensory or motor disorders in the face; photosensitive individuals or those currently taking photosensitive medications; patients unwilling to provide medical history or unwilling to participate in follow‐up.

### 2.2 Equipment

**FIGURE 1:**
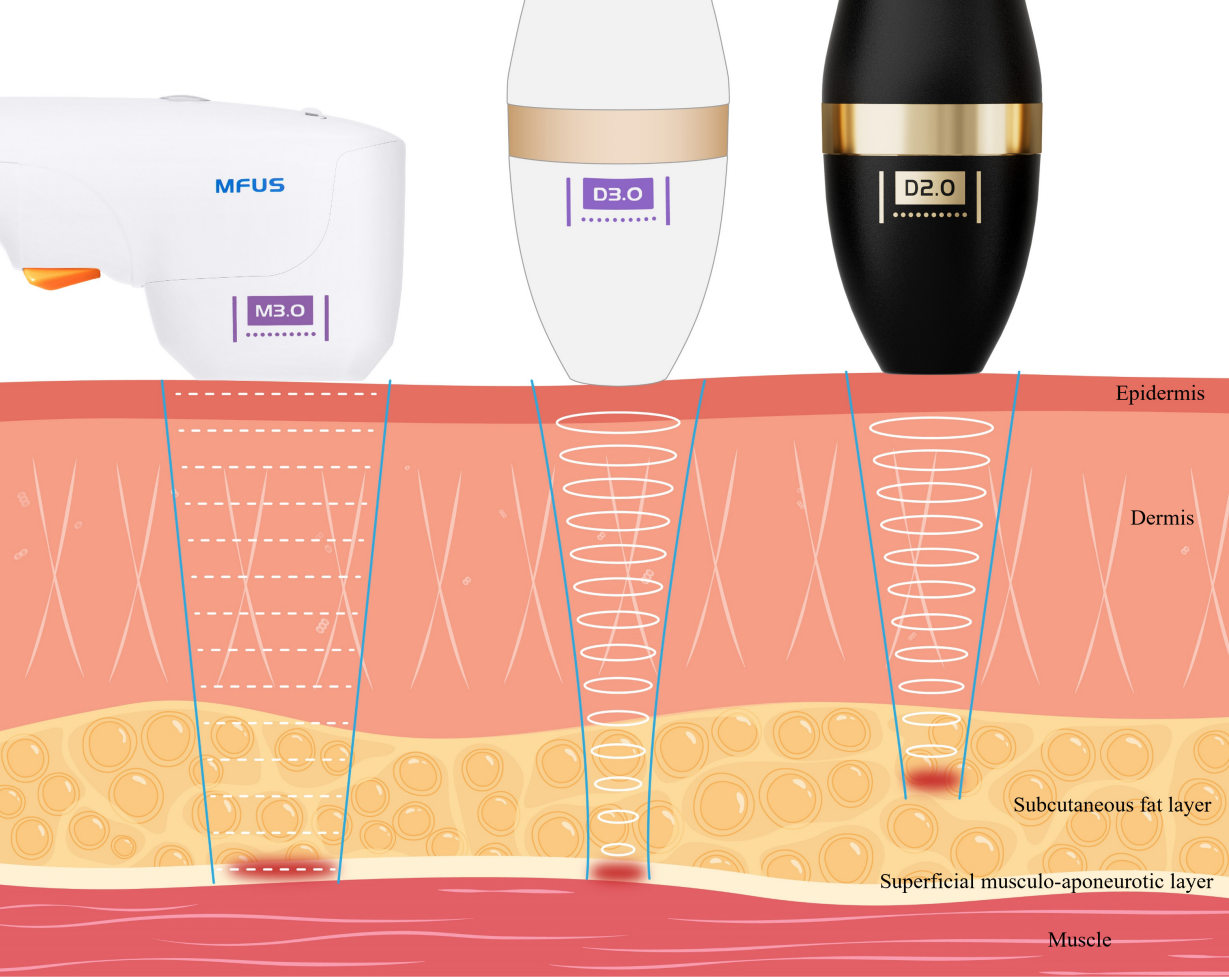
Schematic view of MFUS D/MFUS M applied to skin. Ultrasound beam emanating from probe and penetrating into dermis. The thermal induction zones of MFUS M3.0 and MFUS D3.0 extend to the SMAS layer, with the pulse energy of MFUS D2.0 selectively absorbed by the subcutaneous superficial layer. The MFUS M handpiece emits instantaneous high‐energy beams within the focal area, heating the target tissues in a point/line pattern. The MFUS D handpiece generates a large focal zone of focused ultrasound, concentrating energy on the target tissue layer and gradually heating the planar geometric focal area during sliding superposition treatment.

The MFU ultrasound therapy device utilized in this study was the Micro‐Focused Ultrasound Treatment System (MFUS Pro and MicroUltra; Hunan Peninsula Medical Technology Co, Ltd, China) with two handpieces, microfocused handpiece and dot handpiece. The probes adopted in the study were the microfocused 3.0 transducer (MFUS M3.0) and dot 2.0/3.0 (MFUS D2.0/3.0), configured to generate small (approximately 1 mm^3^) thermal coagulation zones. The thermal induction zone is caused by the selective absorption of focused ultrasound energy within the geometric focal point region of the beam. Adjustable parameters include the source energy from the probe (ranging from 1.32W to 6.63W). The depth and volume/size of thermal injury are determined by the preset focal depth and frequency of the given probe, as well as the inherent characteristics of the treated tissue. The probes used in this study were: MFUS M3.0 (source energy 2.72–3.76W); MFUS D3.0 (source energy 2.27–5.12W); and MFUS D2.0 (source energy 1.5–2.5W) (Figure 1). Compared to low‐frequency probes, high‐frequency probes are associated with shallower tissue thermal effects. During activation and emission, the probe delivers a series of ultrasound pulses.

The photographic equipment used for data collection included a mirrorless camera (SONY ILCE‐7C, SONY China) and a handheld facial 3D imaging system (VECTRA H2, Canfield, USA).

### 2.3 Pretreatment preparation

Before the procedure, participants were instructed to clean their faces. Using a ruler with a minimum scale of 1 mm, the diameter of the irises on both sides was measured. Under consistent lighting conditions, facial measurements were taken by 3D imaging system. Their head positions were adjusted to align with the planes of their eyes and ears, as well as the mid‐sagittal plane, while keeping their gaze directed straight ahead. Frontal and bilateral 45° and 90° photographs of the participants were taken by a digital camera (Nikon‐D60 with a lens of E18.0–55.0 mm f/3.5–5.60SS).

### 2.4 Selection of probes and areas treated

**FIGURE 2:**
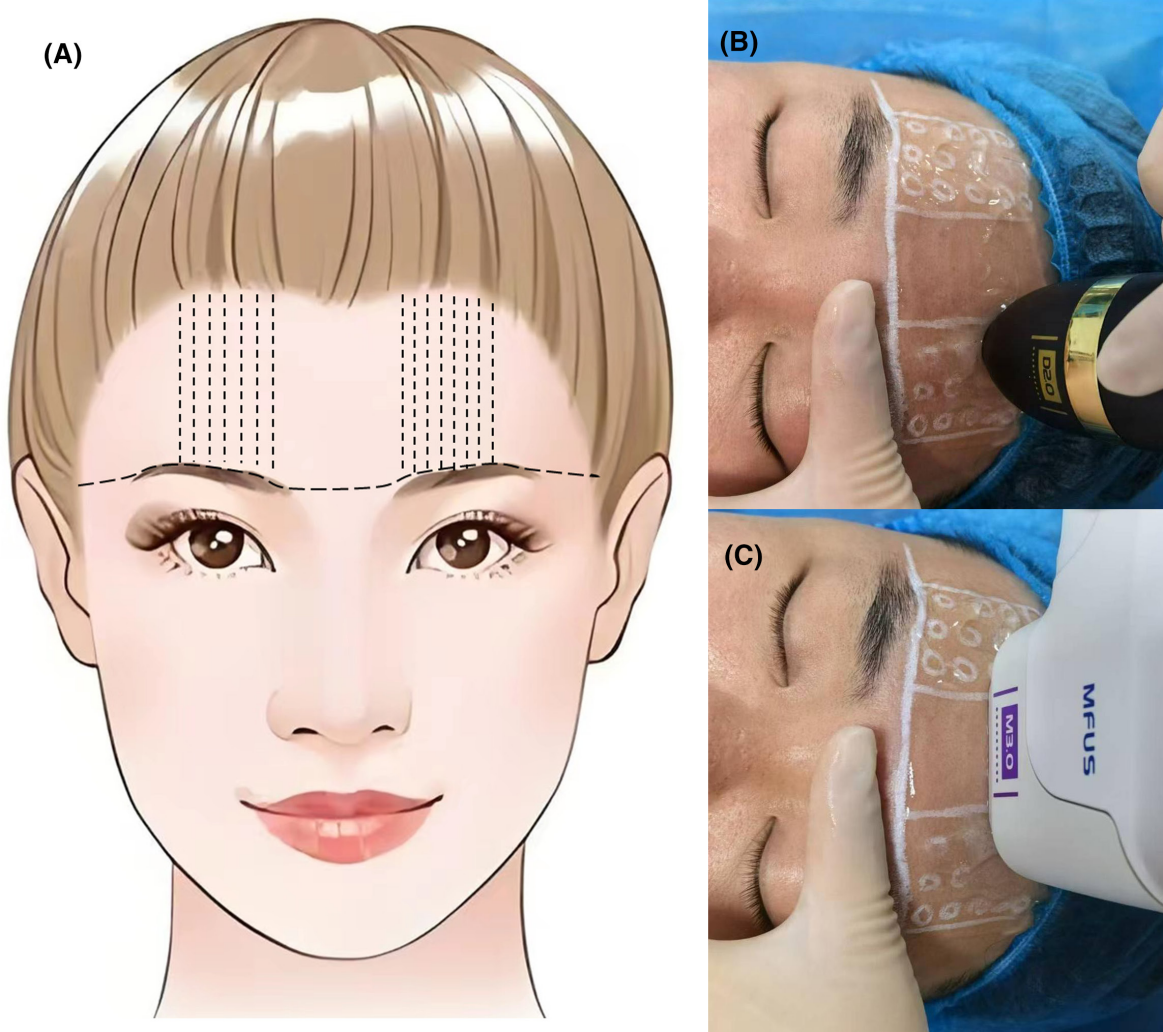
Schematic diagram for treatment area and model selection. (A) The emphasis on important blood vessels and nerves area by dotted line, which treated by (B) MFUS D2.0/D3. (C) MFUS M3.0 was utilized in the remaining skin in the upper facial region.

All patients underwent skin tightening treatment of the upper face using MFU technology. Different probes were selected during treatment according to the location and depth of the target tissue. After cleaning participants' skin, the treatment area on the forehead was marked. Important blood vessels and nerves were emphasized using dotted line (Figure 2A), which were treated by MFUS D2.0/D3.0 (Figure 2B). MFUS M3.0 was utilized to tighten the remaining skin in the upper facial region (Figure 2C). Special ultrasound treatment gel was evenly applied to the skin surface. Prior to placing the treatment handle contact surface on the skin surface, the operators elevated and secured the eyebrows upward, thereby stretching the lax upper eyelid skin (Figure 2B,C). After emitting energy once, the MFUS M handle needed to be moved, with a transfer distance controlled at 2.0–3.0 mm. The MFUS D handle was moved in a steady rotation within the treatment area, ensuring that the handle contact surface was closely adhered to the skin. The two treatment modes complemented each other, with energy controlled at 1.5–2.5 W respectively. For the entire forehead skin, MFUS M treatment emitted 150 pulses, with a time control of 15 min, avoiding the supraorbital nerve and supratrochlear nerve and blood vessel areas. MFUS D emitted continuous pulses for 4000 times over the entire forehead treatment area, with a time control of 5 min. The pulse frequency was adjusted according to the degree of skin aging of each patient. After completion of the procedure, as most patients experienced mild pain and swelling in the treated skin area, soothing repair care was administered to the treated skin area.

### 2.5 Posttreatment care

Immediately after treatment, the ultrasound gel was wiped off, and the face was washed with lukewarm water. Following the digital and 3D photography immediately after the procedure, a mild moisturizing cream was applied to the treated area. Patients were instructed to care for their skin as usual and were not restricted in their normal activities. If necessary, patients could apply medical repair masks for cold compression on the treated area immediately after treatment to minimize local swelling; however, this was not mandatory for all patients as the degree of posttreatment swelling varied. Patients were informed that mild redness and soreness might persist for 1–3 days, and they were advised to contact the research staff immediately if they experienced any other adverse reactions or discomfort.

### 2.6 Outcome measures

#### 2.6.1 Photographic analysis process

**FIGURE 3:**
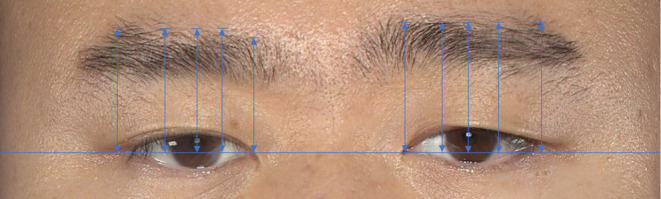
Frontal view of representative patient. The distance between upper edge of eyebrow and the baseline are used to objectively compare brow position in photos before and after treatment.

Measurement of eyebrow elevation: Participants were instructed to look straight ahead naturally with eyes open before taking photos, and the diameter of the participant's iris was measured (in mm) prior to photography. In frontal photographs, measurement lines were drawn by rater‐blinded doctors to calculate the eyebrow height, which included one horizontal line and five vertical lines for each eye. The baseline was formed by connecting the inner canthi of both eyes. The five vertical lines were positioned to pass through the inner and outer canthus points, the inner and outer edges of the iris, and the center of the pupil respectively (Figure 3). The vertical distance between the baseline and the five vertical lines was measured relative to the upper edge of the eyebrow. These data were used to evaluate the change of eyebrow height before and after treatment. Vector analysis using 3D imaging (as indicated by arrows) was employed to observe changes in eyebrow elevation at various time points pre‐ and posttreatment. Objective quantification data on skin movement, including range, intensity, and direction of displacement, is provided through 3D skin vector analysis. Pre‐ and postoperative 3D facial images were captured using the Vectra H2 photogrammetry system (Canfield Scientific Inc.). The Vectra software Mirror Suite (Canfield Scientific Inc.) applied automatic algorithms to generate arrows of varying colors, directions, and lengths, reflecting localized changes in skin displacement. Arrow color transitions from blue, green, yellow, orange, to red as skin displacement magnitude increases, with arrow length proportional to displacement magnitude. These vector arrows indicated motion vectors and skin tightening patterns of the upper facial region above the eyebrows.

Rating scale: This includes pain scores and Global Aesthetic Improvement Scale (GAIS) to evaluate the overall aesthetic improvement after treatment. GAIS scores range from −1 to 3: −1 = worse than the initial state in appearance; 0 = appearance is basically the same as the initial state; 1 = slight improvement in appearance; 2 = significant improvement in appearance; 3 = achieved optimal cosmetic results. The 11‐point visual analog scale (VAS) was used to assess the severity of pain sensation, ranging from 0 to 10, where 0 indicates no sensation and 10 represents the most severe pain imaginable.

### 2.7 Statistical analysis

All data was statistically analyzed using SPSS Version 27.0 (IBM Corp, Redmond, Wash). Continuous variables are described as the mean ± SD, and stratified variables were described as the median ± quartile. The Student's *t*‐test was used to analyze normally distributed continuous variables. the Wilcoxon signed‐rank test was used for the non‐normally distributed continuous variables. *p* < 0.05 was considered statistically significant.

## 3 RESULTS

In 40 subjects, 35 (87.5%) were deemed to have clinically significant brow elevation at 180‐day follow‐up. In the second outcome assessment at the frontal position (straight‐ahead gaze), the median of eyebrow elevation was 2.16 ± 0.63 mm and 1.93 ± 0.57 mm after 90 and 180 days (*p* < 0.01). The effectiveness of the outcomes was more pronounced at 90‐day posttreatment compared to 180 days (*p* < 0.01). The skin in forehead appeared smooth and firm after treatment, with an obvious improvement in the condition of wrinkles.

**FIGURE 4:**
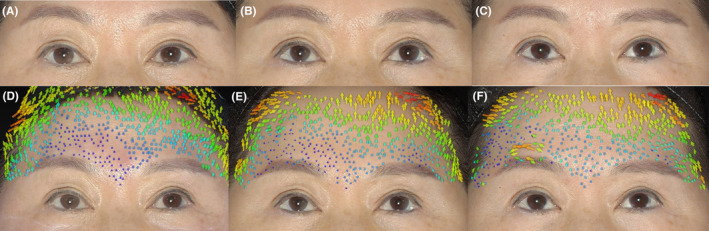
Representative photographs of the results using MFUS D/MFUS M. (A) A 46‐year‐old female patient before treatment, (B) 90 days and (C) 180 days after treatment. Mild elevation was observed in 2D color digital photographs after treatment compared with the state before treatment. (D–F) Three‐dimensional (3D) vector analysis showed that vertical vector displacement of the upper forehead skin appeared (D) immediately after treatment. The vector changes persist at (E) 3 months and up to (F) 6 months postoperatively.

**FIGURE 5:**
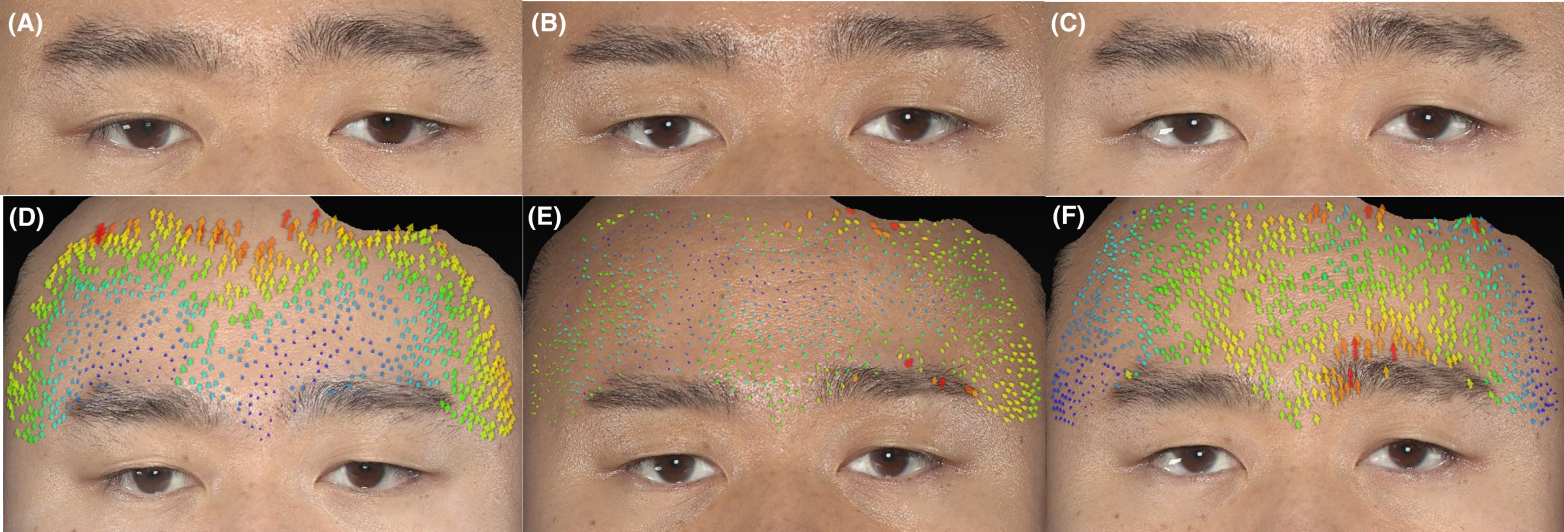
Representative photographs of the results using MFUS D/MFUS M. (A) A 32‐year‐old male patient before treatment, (B) 90 days and (C) 180 days after treatment. Obvious improvement was shown in 2D photographs after treatment. D‐F 3D vector analysis showed that the vertical vector changes are predominantly concentrated in the upper‐middle region of the forehead (D) immediately after treatment and (E) after 3 months. (F) After 6 months, vertical vector changes are more pronounced in the middle of the forehead and the inner side of the eyebrows.

In 3D skin vector analysis, three types of displacement vectors of varying lengths on the forehead skin of all subjects were observed. They presented as vertical vectors (with an average angle of 90° ± 5° relative to the *x*‐axis) and lateral oblique vectors (with an average angle of 95–175 or 5–85 relative to the *x*‐axis), with less frequent parallel oblique vectors (angles ranging from 0 to 5 or 175 to 180 relative to the *x*‐axis) (Figures 4 and 5). No downward vectors were detectable.

In the follow‐up after the procedure, the GAIS scores of both the doctors and the participants improved. In the follow‐up after the procedure, 28 participants (70%) received a GAIS score of 2 after 90 days, and 22 participants (55%) received a GAIS score of 2 after 180 days. After 90 days, 33 cases (82.5%) and after 180 days, 28 cases (70%) received a GAIS score of 2 from the investigators. All participants presented a transient slight (1–2 on a 4‐point scale) erythema and edema immediately after treatment. The average VAS was 2.41 ± 0.70 (range from 1.5 to 3.5). Following standard posttreatment care, these symptoms alleviated and resolved during the 7‐day‐follow‐up. Other complications, including ulcerations, bleeding, nerve/muscle of dysfunction and hyperpigmentation were not shown in these participants. Pain scores for operations were 3–5 on a 10‐point scale (Table 1).

**TABLE 1:** Participant characteristics and outcome evaluation.

	Mean‐SD (median) or *n* (%)	*p*‐value (95%CI)
Gender		
Female	35 (87.5%)	
Eyebrow elevation		
90‐day follow up	2.16 ± 0.63 mm	< 0.01
180‐day follow up	1.93 ± 0.57 mm	< 0.01
VAS	2.41 ± 0.70	

Abbreviations: CI, confidence intervals, VAS, visual analog scale.

## 4 DISCUSSION

Ultrasound operates by converting sound energy into heat through mechanical vibration and molecular friction, thereby raising the temperature of the tissues. Overall, the selective coagulation changes induced by ultrasound are achieved within the focal zone of the beam, with no significant impact on tissues near or distant from the focal zone. Focused ultrasound is a widely used technology in clinical applications, focusing on specific areas of the body (target tissues) while minimizing the risk of damage to surrounding healthy tissues.^8^ High‐intensity focused ultrasound (HIFU) is similar to ultrasound imaging but is less invasive. This technique uses lower frequencies, reducing the common side effects associated with other treatment methods.^9^, ^10^, ^11^ The characteristics of this ultrasound energy increase its applicability for skin tightening. Ultrasound therapy is a method of skin rejuvenation and tightening using MFU technology. Initially, ultrasound delivers energy to the deeper layers of the face, including the SMAS, which is most effective for inducing skin tightening and lifting.^12^, ^13^ Moreover, by avoiding secondary scattering and absorption in the epidermis and dermis layers, the risk of accidental skin damage can be reduced. In addition to ionizing radiation, ultrasound is the only technology capable of selectively delivering energy deep into target tissues. Therefore, focused ultrasound is a safe and effective method for facial skin tightening.

Facial rejuvenation in the forehead and temple areas requires attention to detail. The forehead skin is thin and vascular, necessitating a thorough understanding of the structural relationships between blood vessels, nerves, and soft tissues during phototherapy, ultrasound, and other rejuvenation treatments in these areas. The forehead skin consists of thin, dense, and resilient tissues, receiving rich blood supply from various arteries such as the superficial temporal artery, supraorbital artery, and zygomatic artery.^14^, ^15^ Therefore, during ultrasound treatment, it is essential to avoid or minimize the areas where the supraorbital and zygomatic nerves traverse. Compared to probes M4.5 and D4.5, the energy accumulation of treatment heads M3.0, D3.0, and D2.0 is shallower, suitable for areas with thinner forehead skin tissue. Especially, the D3.0 and D2.0 probes can more accurately guide energy to shallower subcutaneous tissues, avoiding injury to the dermal papillary layer, which is prone to damage.

This study conducted MFU treatment on the forehead and bilateral temple areas of 40 patients, resulting in an average increase in brow height of 2.1 mm. Over 70% of treated patients achieved this effect, which was maintained for 3 months posttreatment. Side effects were limited to transient erythema, which is common in all laser or intense pulsed light, radiofrequency, and other treatments.

When evaluating candidates for upper facial rejuvenation surgery or nonsurgical treatments, several factors need to be considered: presence of brow ptosis, degree of skin laxity, thickness of subcutaneous fat, morphology of upper eyelid folds, types of periorbital wrinkles, and the patient's age. Proper assessment of whether patients are suitable for MFU treatment is a prerequisite for achieving good efficacy and patient satisfaction. When addressing aging issues in the upper face, including upper eyelid skin laxity, it is crucial to comprehensively evaluate and improve the aging status of the periorbital area. Careful assessment and examination of brow position are essential to estimate whether concurrent brow lift treatment is required to achieve overall aesthetic rejuvenation of the periorbital area. In young females, the normal position of the brow is approximately 5 mm above the orbital margin, while in males, the brow position is lower and often at the level of the orbital margin.^1^, ^16^, ^17^ For mild to moderate brow ptosis, using the MFU facial treatment head to cover the forehead and lateral orbital areas while assisting in fixation, lifting, and traction of the periorbital and temple regions can enhance the immediate tightening effect and improve brow ptosis. However, for severe brow ptosis, brow lift surgery is recommended for correction. Limited correction is difficult for concurrent upper eyelid skin laxity, which can be addressed by MFU combined with upper eyelid skin laxity correction surgery/upper eyelid shaping surgery to avoid post‐brow lift eyebrow loss, scarring, and exacerbation of brow ptosis. Noninvasive focused ultrasound skin tightening is expected to reduce wrinkles and sagging in the shortest downtime without leaving scars or serious adverse effects. The author recommends repeat treatments at intervals of 6–8 months.

Similar to intense pulsed light, lasers, and radiofrequency, MFU is one of the most commonly used noninvasive methods for skin tightening in China. However, treatment outcomes are influenced by individual differences such as skin type, age, and treatment site, resulting in variations in treatment efficacy for each person. Based on the work and prospective feasibility studies reported in this paper, it is hoped that more precise and less adverse reaction MFU upper facial rejuvenation treatment plans will be provided to patients.

### 4.1 Limitations

This study has limitations, notably a small sample size with 40 participants, of which 37 were female subjects. We plan to assess the effectiveness of this technology in male subjects in future studies. Additionally, the lack of standard measurement qualifying upper skin laxity was the other limitation. We assessed brow height to evaluate the rejuvenation of the upper facial skin based on the participants' complaints. The subjective method will be adapted to evaluate the improvement of overall skin laxity.

## 5 CONCLUSIONS

Our study demonstrated that focused ultrasound could enhance the rejuvenation of upper facial skin in a safe and effective manner. A solitary focused ultrasound treatment on the forehead raises the brows by approximately 2.1 mm.

## AUTHOR CONTRIBUTIONS

Chen Wei had full access to all of the data in the study and took responsibility for the integrity of the data and the accuracy of the data analysis. Cai Wei were involved in the study concepts and design. All authors were involved in the acquisition, analysis, and interpretation of data. Yuequ Deng and Guanqun Qiao finished the analysis and drafted the manuscript. All authors read, critically revised, and approved the manuscript. All the named authors meet the International Committee of Medical Journal Editors (ICMJE) criteria for authorship for this article, and take responsibility for the integrity of the work as a whole and have given their approval for this version to be published.

## FUNDING INFORMATION

No funding was received for the work presented in this article.

## CONFLICT OF INTEREST STATEMENT

This study has no competing financial interests exist.

## ETHICS STATEMENT

This study was approved by the Ethics Committee of The Second Affiliated Hospital of Nanjing Medical University and have been performed in accordance with the ethical standards as laid down in the 1964 Declaration of Helsinki and its later amendments or comparable ethical standards.

## CONSENT

Informed consent was obtained from all individual participants included in the study.

## Data Availability

The datasets generated and/or analyzed during the current study are not publicly available due to privacy and ethics but are available from the corresponding author on reasonable request.
